# VMAT-based total body irradiation on a conventional LINAC: workflow, procedure and preliminary results

**DOI:** 10.1007/s12094-026-04245-4

**Published:** 2026-02-17

**Authors:** I. Rey López, A. A. Alayón Afonso, M. P. Melián Jiménez, C. Madán Rodríguez, A. Díaz Martín, M. Sánchez Carrascal, E. Ruiz Egea, M. Lloret Sáez-Bravo

**Affiliations:** 1 Department of Radiation Oncology, Dr Negrín University Hospital of Gran Canaria, Las Palmas, Spain; 2 Department of Medical Physics, Dr Negrín University Hospital of Gran Canaria, Las Palmas, Spain

**Keywords:** Total body irradiation, TBI, Volumetric modulated arc therapy, VMAT, Conventional LINAC, Procedure, Workflow

## Abstract

**Background/Purpose:**

The aim of this study was to evaluate the feasibility of implementing volumetric modulated arc therapy (VMAT)–based techniques and extended CBCT image guidance for total body irradiation (TBI) treatment using a conventional linear accelerator.

**Methods:**

Patients eligible for TBI between November 2016 and December 2024 were included in the analysis.

Patients received a total dose of 4–12 Gy, given in six fractions within 3 days, two fractions/day with 6 h minimum interval between fractions, or 2 Gy in one fraction, depending on the clinical indication.

During the initial phase of the protocol, PET–CT imaging was used to obtain full-body CT datasets. Subsequently, CT simulation was performed using a multislice Siemens CT scanner available in the Radiation Oncology Department. In this setting, two CT studies were acquired per patient, one extending from the pelvis to the head (upper scan) and a second from the pelvis to the feet (lower scan), which were merged into a single dataset for treatment planning.

Dosimetric planning was performed using a multi-isocenter approach with the Eclipse™ treatment planning system, employing volumetric modulated arc therapy (VMAT) to achieve the prescribed dose distribution. In the initial stage of the treatment program, treatments were delivered on a Varian CLINAC DHX linear accelerator. Following its decommissioning, treatment delivery was transitioned to Varian TrueBeam linear accelerators (models SN3790 and SN2137). Treatment delivery, including verification and patient positioning, was performed sequentially, beginning with the upper body followed by the lower body. Image guidance was initially based on kV–MV imaging and was later replaced by extended-field cone-beam computed tomography (CBCT), which was registered to the simulation CT to enable automated setup corrections. Dosimetric parameters and setup verification metrics were subsequently analyzed.

**Results:**

Between November 2016 to December 2024, 27 patients fulfilled the inclusion criteria. All scheduled sessions were completed, amounting to a total of 148 treatment fractions. The average number of isocenters used to generate the treatment plans was 7,11 (6–12). The mean lung dose was 10.12 Gy (range 8.97–11.07 Gy). Dose homogeneity achieved across all sessions was 1.24 (1.11–1.41). After image acquisition, mean setup corrections were 0.06 cm lateral (range 0.00–2.00 cm), 0.26 cm vertical (0.00–2.00 cm), and 0.03 cm longitudinal (0.00–1.30 cm) in head-first plan. Laterally, 0.26 cm (range: 0.00–2.00 cm) vertically, and 0.03 cm (range: 0.00–1.30 cm) longitudinally in feet first plan. The average duration of each session, from the first image acquisition to the completion of the final field, was 87 min (range 60–159).

**Conclusions:**

Our study demonstrates that VMAT-based TBI is a feasible and promising alternative to conventional 2D-TBI, providing improved dose homogeneity, enhanced organ sparing and with reproducibility comparable to previously reported HT systems. These findings support the integration of VMAT techniques on conventional LINACs for TBI treatments, although further prospective studies are needed to confirm long-term clinical benefits.

## Introduction

Total body irradiation (TBI) involves the delivery of a radiation dose encompassing the entire body volume and remains a well-established component of conditioning regimens before hematopoietic stem cell transplantation (HSCT), particularly in acute lymphoblastic leukemia (ALL) and acute myeloid leukemia (AML) [1–9]^.^

This treatment is performed with three main objectives. First, to eradicate residual neoplastic cells throughout the body and eliminate tumor cell clones resistant to chemotherapy, particularly in sanctuary sites where chemotherapy has limited efficacy. Second, to eliminate malignant cells from the bone marrow, thereby creating space for subsequent repopulation with donor cells. Finally, to provide a sufficient degree of immunosuppression, reducing the likelihood of host immune cells rejecting the donor graft and lowering the incidence of graft-versus-host disease (GVHD) [1, 2].

Over time, TBI has evolved in terms of total dose, fractionation, and technique. Hyperfractionated myeloablative regimens typically deliver 12–13.5 Gy in 6 fractions of 2–2.25 Gy, administered twice daily over three days [3, 4]. For purely immunosuppressive intent, lower-dose regimens (< 8 Gy) or single-fraction schedules (< 5 Gy) are used, especially in patients with marrow aplasia or those unsuitable for high-dose TBI due to comorbidities or age [2]. Retrospective series suggest that myeloablative TBI can improve overall survival (OS) despite higher pulmonary toxicity [10, 11]. Nevertheless, dose escalation has been associated with lower relapse rates, but with a concomitant increase in treatment-related mortality, particularly due to complications such as pulmonary fibrosis and hepatic veno-occlusive disease [1].

Traditional TBI techniques use 2D or 3D open-field irradiation, with the patient seated on a specialized support or in lateral decubitus at an extended source-to-skin distance (SSD) [5, 6, 12]. This ensures full-body coverage with field sizes of approximately 70 × 200 cm^2^.

Two opposing fields (AP and PA) are used. Each fractionated dose delivered in two equal parts, changing only the patient’s position. The dose is prescribed to a specific point in the body (the prescription point). The entire body must receive a dose within ± 10% of the prescribed dose at this point.

To eliminate the skin-sparing effect of high-energy photons, a plexiglass barrier or a low-density beam spoiler is placed near the patient. This ensures that 90% of the prescribed dose is delivered to the skin surface [7]. Treatment planning is done via CT in the supine position. However, this differs from the treatment position, introducing dose distribution uncertainty. Positioning is verified before each session using light field alignment and lung shielding checks in the anterior field, without the use of image-guided radiotherapy (IGRT).

This technique has several limitations. Prolonged treatment session times, limited ability to spare organs at risk, low beam conformity, and dose heterogeneity. Some studies document dose variability exceeding 10–20% [2, 7, 10], which contributes to increased acute and late toxicity.

A critical limitation is the dose received by the lungs. This issue arises from the commonly used single-point dose calculation model. Dose-volume histograms (DVHs) are rarely available for patients treated with this technique. As a result, a detailed assessment of pulmonary dose tolerance is not possible [13]. It should be noted that the reported toxicity is based on measurements obtained at the designated calculation points. Given the inherent technical limitations of dose distribution modeling, it is reasonable to assume that the actual heterogeneity may be significantly greater.

To address these uncertainties and following the recommendations of international guidelines [2], recent years have seen a shift toward optimized techniques. These include CT-based 3D simulation and treatment using intensity-modulated radiotherapy (IMRT) or volumetric modulated arc therapy (VMAT) through inverse planning. The aim is to improve dose uniformity and target coverage while minimizing dose exposure to organs at risk [8, 9].

The first TBI treatments using optimized techniques were implemented with helical tomotherapy (HT) systems starting in 2005 at European and American centers [14, 15]. HT combines a LINAC with a CT-based gantry, enabling continuous helical delivery that avoids field junctions and provides highly conformal dose distributions.

Initially, these treatments were applied to pediatric populations, as reported by Wong JY et al. [15], and later extended to adult patients, as demonstrated by the work of Zhang-Velten, E.R. et al. [16], among others. For the first time, this approach enabled the delivery of highly conformal doses to large volumes, covering treatment lengths of approximately 160 cm [8], eliminating the need for field junctions and potential overlaps. This ensured lower doses to healthy organs and improved toxicity outcomes [17, 18].

To extend this technique to centers without HT systems, several groups have reported the use of VMAT on conventional linear accelerators (LINACs) [16, 19–21]. Unlike tomotherapy, which employs a continuous helical approach, VMAT delivers the dose through discrete arcs.

The objective of this study was to describe the VMAT-TBI protocol implemented in our department and to present preliminary results on treatment homogeneity, reproducibility, and resource utilization.

## Material and methods

A retrospective analysis was conducted on patients treated with total body irradiation (TBI) at the Department of Radiation Oncology, University Hospital of Gran Canaria Dr. Negrín, between November 2016 and December 2024. Our hospital is the reference center for bone marrow transplantation in the Autonomous Community of the Canary Islands.

The inclusion criteria were: patients aged ≥ 18 years who were eligible for bone marrow transplantation in the first or second relapse of their hematologic disease with an indication for TBI. Both myeloablative regimens (for acute lymphoblastic leukemia, acute myeloid leukemia, chronic myeloid leukemia, and non-Hodgkin lymphoma) and myelosuppressive regimens (for aplastic anemia) were included.

Patients were referred from the Hematology Department through formal consultation. Once the indication and timing were confirmed, the workflow began: a combined CT and simulator slot was booked at least three weeks before treatment, and the accelerator nurse scheduled TBI sessions on the linear accelerator. The Medical Physics Department was also informed. After medical evaluation and explanation of the procedure, the patient signed the informed consent. Treatment preparation could be conducted on an outpatient basis.

The total dose and treatment duration varied according to the intended approach. Myeloablative regimens consisted of 12 Gy delivered over three consecutive days, in two daily fractions of 2 Gy separated by at least six hours. Myelosuppressive regimens consisted of 2–4 Gy delivered in a single day. Radiotherapy was always performed prior to intensification chemotherapy.

From 2016 to 2022, all treatments included in this study were delivered on a Varian CLINAC DHX linear accelerator, with a beam-matched system available in case of equipment failure. Following the decommissioning of one of the CLINAC DHX beam-matched units in 2022, treatments included in the study were subsequently delivered on a Varian TrueBeam linear accelerator (model SN3790), for which a dosimetrically matched system (model SN2137) was also available.

### Positioning and planning image acquisition

During the initial phase of the program, full-body CT images for treatment planning were acquired using the hospital’s PET/CT system in the Nuclear Medicine Department, as the CT scanner in the Radiation Oncology Department did not allow imaging of the entire body length in a single acquisition. Subsequently, following the availability of tools enabling the concatenation of images from multiple acquisitions (2024), CT simulation was performed using a multislice Siemens Somatom Confidence CT scanner (Siemens Medical Solutions USA, Inc.) [22] in the supine position.

For patient immobilization, a carbon fiber board with indexing bars was used, on which a vacuum cushion was placed and molded from the axillae to the feet, conforming as closely as possible to the patient’s anatomy. Proper alignment was ensured by stabilizing the pelvis and aligning the midline of the forehead and chin with the sternum, pubis, and legs.

To immobilize the head and neck, a long thermoplastic mask extending to the shoulders was used, maintaining these structures in a neutral position aligned with the thorax. The mask was removed during treatment of the lower body to improve patient comfort.

The arms and hands were positioned along the sides of the body, and the legs were kept together. A tubular elastic mesh (Tubifix^R^) was applied around the thorax (including arms) and legs to enhance immobilization.

Reference marks for laser alignment were placed on the patient’s pelvis and on the vacuum cushion at the same level. Additional midline marks were drawn on the mask (forehead and chin) and replicated on the vacuum cushion, as well as on the thorax, pelvis, and feet.

For CT simulations performed in the Radiation Oncology Department, image acquisition was carried out using two sequential scans per patient, with the pelvis used as the reference point. First, a head-first supine (HFS) acquisition covering the upper body was obtained (craniocaudal scan). As a rotating treatment couch was not available at our institution, the patient was then carefully assisted off the couch, and all immobilization devices were repositioned and re-indexed to ensure identical alignment. The patient was subsequently repositioned 180° in a feet-first supine (FFS) orientation (feet-to-gantry) to acquire the lower body images (caudocranial scan), thereby maintaining reproducibility between both setups.

CT simulation was performed with a slice thickness of 5 mm.

After completion of both CT scans, feasibility and collision-free positioning were verified on the linear accelerator. If a potential collision was detected, the reference point was adjusted accordingly.

### Planning imaging preparation

As described in the previous section, initial treatment planning relied on full-body CT datasets acquired in a single imaging session. Following the transition to CT simulation performed within the Radiation Oncology Department using multiple acquisitions, the development and validation of a CT concatenation strategy became necessary, as the maximum longitudinal scan length of the available CT scanner did not allow whole-body imaging in a single acquisition.

Accordingly, the superior (head-first supine, HFS) and inferior (feet-first supine, FFS) CT datasets were processed to generate a single concatenated CT dataset suitable for treatment planning and dosimetric calculations. This approach ensured complete anatomical coverage while preserving the original Hounsfield Unit (HU) information from the acquired images.

During the early implementation phase, CT concatenation was achieved through an iterative and labor-intensive workflow in which several approaches were evaluated. These included open-source software (3D Slicer) [23] and commercial platforms not specifically designed for automated whole-body CT stitching, such as Velocity™ (Varian Medical Systems Palo Alto, California) [24]. Although technically feasible, these methods required extensive manual intervention and time-consuming quality control procedures to verify anatomical continuity at the junction region, registration accuracy, and consistency of HU values between datasets. At the time this study was conducted, we were exploring alternative approaches to achieve this fusion, such as the TXIhelper script [25]^.^

### Contouring

The Eclipse™ Treatment Planning System (TPS) (Varian Medical Systems, Palo Alto, California) was used for contouring. A semi-automatic contouring tool within the software was employed to delineate the following structures: skin, lungs (right, left, and combined), kidneys, and lenses, followed by manual adjustments when required.

The planning target volume (PTV) was defined by subtracting a 5 mm inner margin from the skin to improve treatment plan homogeneity. The lungs were delineated individually, excluding a 5 mm internal margin, and a combined lung volume was generated for dosimetric analysis. Kidneys and lenses were completely excluded from the PTV, with no additional margins applied.

To minimize inter-operator variability in image fusion and contouring, all TBI planning was performed by a small, specialized team comprising two radiation oncologists and four medical physicists, following predefined contouring templates and fusion protocols. This team was gradually established over the course of the study period.

### Dosimetry

Treatment plans were generated using the VMAT technique with RapidArc software integrated within the Eclipse™ TPS. All plans employed 6 MV photon beams.

Dosimetry consisted of two plans created on the same full-body CT dataset: a superior plan in the head-first supine (HFS) position and an inferior plan in the feet-first supine (FFS) position. The superior PTV extended from the head to the proximal femur, encompassing the pelvis (an area of high bone density) to ensure adequate coverage and to prevent underdosage or overdosage (cold/hot spots) in the junction region of the two CT scans. The inferior PTV extended from the proximal femur to the feet.

**Fig. 1:**
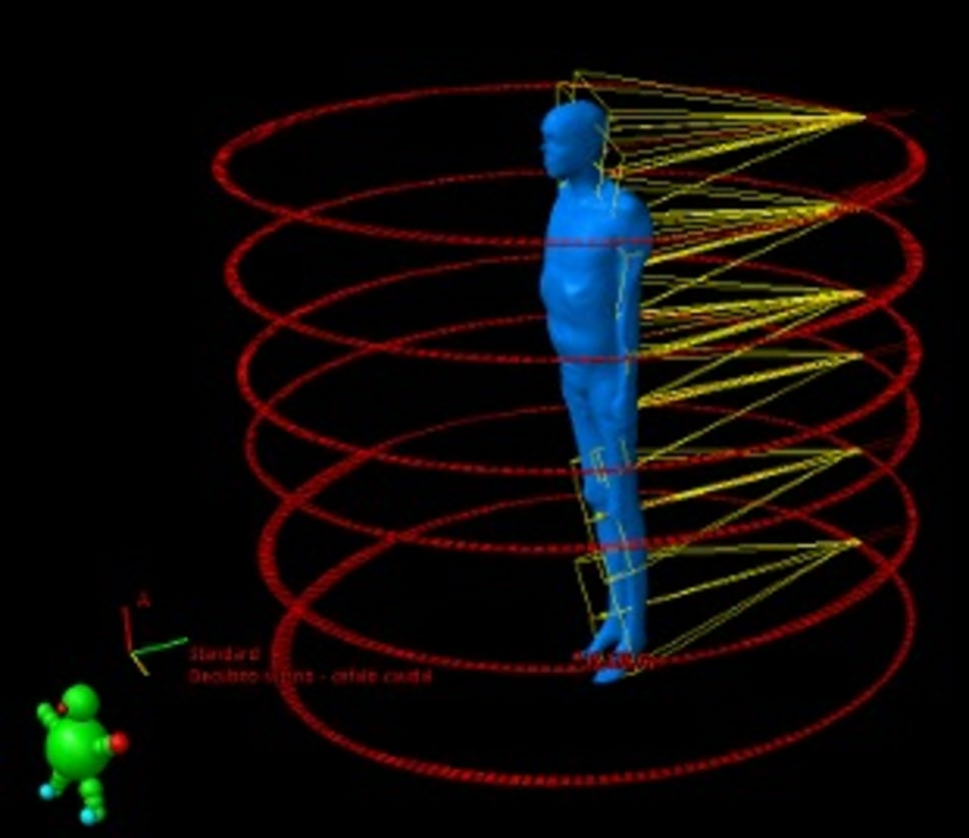
Example of a treatment plan with multiple isocenters

For planning, several equidistant isocenters were distributed along the longitudinal axis, originating at the pelvis and extending cranially and caudally. A multi-isocentric planning approach was employed. The superior plan was optimized first, followed by the inferior plan using the *Base Plan* option (Fig. 1).

The number of isocenters depends on the patient height, maximum field size, and collimator angle. Each isocenter included 2–4 VMAT arcs. On the Varian CLINAC DHX LINAC, equipped with a standard Millennium MLC (40 × 40 cm^2^ field size), fewer fields per isocenter were required, with inter-isocenter distances of approximately 30–35 cm, thereby reducing the overall number of isocenters.

The Varian TrueBeam linacs in our department, equipped with Varian high-definition MLCs (field size 22 cm in Y and 40 cm in X), required a different strategy. To maximize longitudinal coverage and modulation, two opposed collimator angles (typically 30–50° and 330–310°) were used. Each collimator orientation was further split into two sub-fields (y1 = y2 = 11 cm; × 1 = 20 cm with × 2 = 0 cm, and vice versa) to minimize leaf overtravel. This configuration provided approximately 30–35 cm of longitudinal coverage per isocenter with sufficient modulation to achieve clinical goals. For example, in a patient measuring 180 cm, six isocenters spaced 25–30 cm were typically required.

All isocenter groups within each plan maintained identical lateral and vertical coordinates, restricting movements between isocenters to the longitudinal axis. Beam arrangements were then configured and optimized to meet the dosimetric objectives for PTV coverage (Table 1).

**Table 1:** Dosimetric objectives for PTV coverage and OAR sparing (12 Gy schedule)

Summary of clinical goals		
Structure	Priority	Objetive
PTV	P1	D95% > 11.40 Gy
	P1	D2.0% < 12.80 Gy
Kidneys	P1	Dmin > 8 Gy
	P2	Dmed < 10 Gy
Lens_L	P2	Dmax < 8 Gy
Lens_R	P2	Dmax < 8 Gy
Lungs	P1	Dmin > 8 Gy
Lungs-0,5	P2	Dmed < 10 Gy

### Dose-volume histogram (DVH) Analysis

DVH analysis was performed for patients treated with the 12 Gy myeloablative schedule. Dosimetric objectives were defined for the planning target volume (PTV) and organs at risk (OARs) and were classified into two levels of priority: P1 = mandatory objectives for plan acceptance, and P2 = secondary objectives, applied when feasible to further optimize organ sparing.

## Treatment planning evaluation and approval

**Fig. 2–5:**
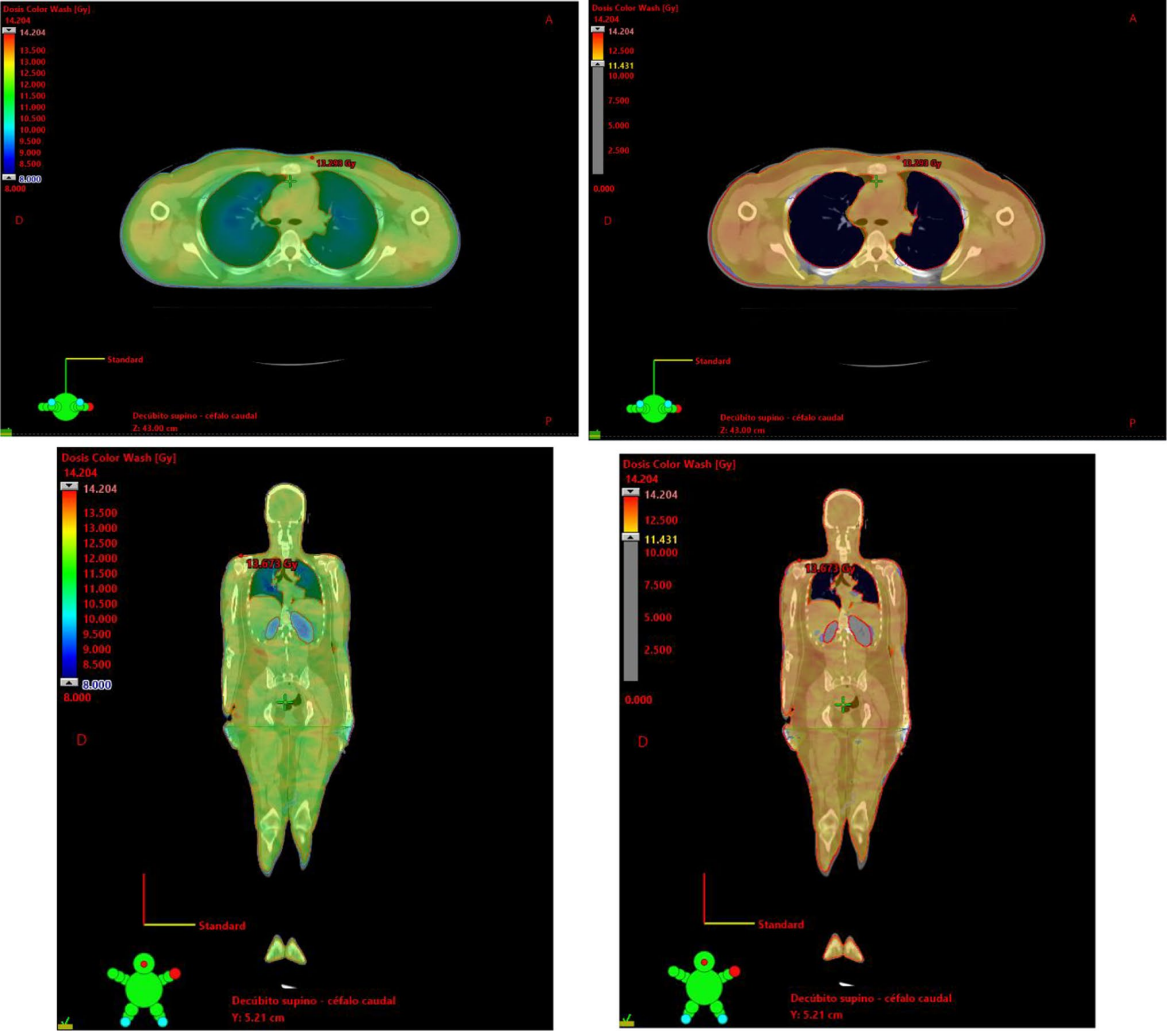
Figures 2 and 3 show the distribution of the 8 Gy dose in the coronal and axial slices. Figures 4 and 5 show the distribution of the 95% dose, which corresponds to 11.40 Gy

Treatment plans were reviewed to ensure that 95% of the PTV received at least 95% of the prescribed dose, in accordance with ICRU Report 83 recommendations [26, 27]. Dose-volume histograms were systematically assessed prior to plan approval. Once the planning was verified, it was approved for clinical use (Figs. 2–5).

### Pre-treatment quality assurance

Following treatment planning and dosimetric optimization, pre-treatment quality assurance (QA) was performed according to standard VMAT protocols. An ArcCHECK phantom (Sun Nuclear, Melbourne, FL) was used to verify treatment delivery reproducibility. An extended CT dataset of the phantom was acquired to simulate the complete treatment and calculate the expected dose distribution.

All treatment fields were subsequently delivered to the phantom in accordance with large-field QA procedures. This involved sequential irradiation of the superior and inferior halves of the patient’s treatment fields, followed by merging the corresponding measurements to reconstruct the full dose distribution.

Measured and calculated dose distributions were compared using the gamma index method (3%/3 mm, global normalization, absolute dose). QA was performed according to the recommendations of the AAPM Task Group 218 report [28], with an acceptance threshold of a gamma passing rate > 97%.

### Administration of TBI treatment. Daily positioning and image control in the unit

Patients were admitted to the hospital at least one day prior to the initiation of radiation therapy and remained hospitalized until completion of the transplantation process. Before each treatment session, Ondansetron (4 mg) was administered, and patients underwent a 4-h fasting period. Besides this treatment-specific prophylaxis, patients were allowed to continue their concomitant medications during hospitalization.

Treatment was delivered in two halves, starting with the upper PTV after verification of patient positioning. Image guidance was performed as part of an IGRT protocol.

Initially, IGRT was based on kV/MV imaging. Images were acquired at each isocenter from the pelvis to the head to complete the upper PTV. Each image was registered to the digitally reconstructed radiograph (DRR) obtained from the simulation CT. At each isocenter, longitudinal, vertical, and lateral displacements were measured and recorded but not immediately applied. After all images had been acquired, the average displacement in each direction was calculated and applied, and treatment was then delivered in the reverse direction (from head to pelvis). For subsequent isocenters, only the predetermined longitudinal shift was applied, while vertical and lateral coordinates remained unchanged. The same process was repeated for the lower PTV, with imaging performed from the pelvis to the feet.

**Fig. 6:**
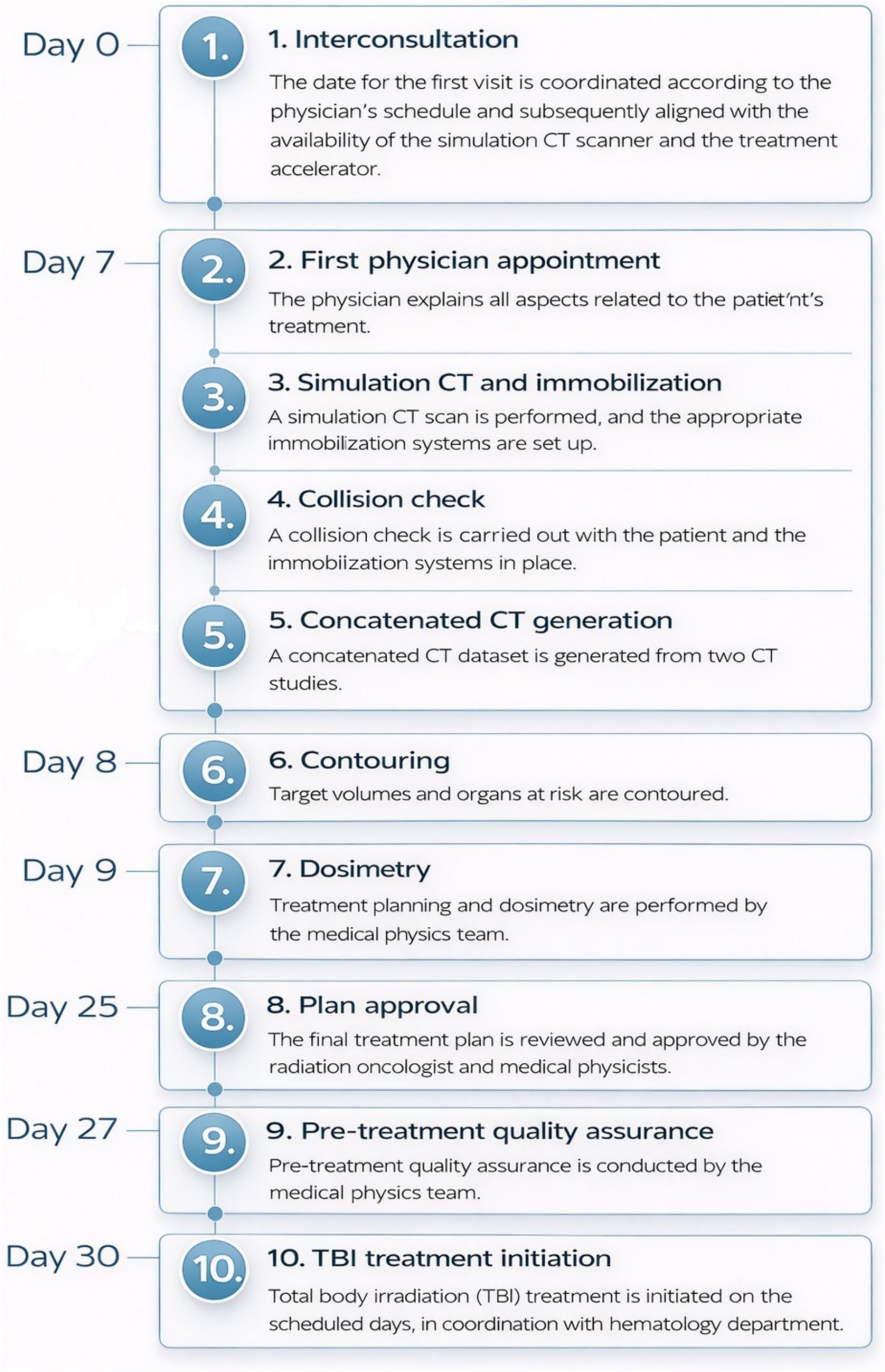
Workflow of Total Body Irradiation in our Department

From 2023 onwards, IGRT was optimized with the integration of extended cone-beam computed tomography (CBCT). Treatment continued to be delivered in two halves, beginning with the upper PTV. An extended CBCT covering the entire PTV was acquired and fused with the planning CT to perform automatic registration. This approach reduced setup time, minimized manual intervention, and improved reproducibility (Fig. 6).

## Statistical analysis

The study variables included demographic characteristics (age at the time of treatment and sex), hematological diagnosis, radiotherapy-related toxicity, disease relapse, and overall survival. Dosimetric variables analyzed were the number of isocenters, homogeneity index (HI), organ-at-risk (OAR) doses in patients treated with 12 Gy schedules, the IGRT technique used, and session duration.

The homogeneity index (HI) was defined as D2%/D98%, with values closer to 1.00 indicating better dose uniformity. OAR dose analysis was restricted to patients who received the 12 Gy regimen. Session duration was calculated from the acquisition of the first CBCT to the last beam-off, including couch shifts and patient rotations.

Statistical analysis was conducted using SPSS Statistics for Windows, Version 28.0 (IBM Corporation, Armonk, NY) [29].

## Results

In this period, a total of 27 patients were included. Patients were recruited from 5 hospitals in the Canary Islands, mainly from the University Hospital of Gran Canaria Dr. Negrín (19 patients, 70.37%).

Mean age of the patients was 36.7 years (range: 15–59 years), 80% of them being male. The most common diagnosis was B-cell acute lymphoblastic leukemia (ALL) (66.7%), followed by T-cell ALL (11.1%) (Table 2).

**Table 2:** Patient and disease characteristics

	*n* (%) / Mean (range)
Age (years)	36.7 (15–59)
Sex	Male: 20 (74.1%) Female: 7 (25.9%)
Diagnosis	B-cell ALL: 18 (66.7%) T-cell ALL: 3 (11.1%) Others: 6 (22.2%)

A total of 148 treatment sessions were administered to the 27 patients. The most common fractionation regimen was 12 Gy in 2 daily fractions of 2 Gy, prescribed to 24 patients (88.9%). One patient received 2 fractions of 2 Gy in a single day, and two patients received a single 2 Gy fraction as part of a myelosuppressive regimen.

### Dosimetry

Treatment plans involved a mean of 7,11 isocenters (range, 6–12), each comprising between 2 and 4 full VMAT arcs.

For patients receiving the 12 Gy hyperfractionated regimen (6 × 2 Gy), the average dose distribution was D2% = 13.32 Gy (range, 12.90–14.76 Gy), D98% = 10.89 Gy (range, 9.69–12.60 Gy), and D50% = 12.55 Gy (range, 11.95–13.84 Gy).

For the 4 Gy schedule, values were D2% = 4.23 Gy, D50% = 4.04 Gy, and D98% = 3.75 Gy (no variation observed).

For patients treated with a single 2 Gy fraction, the mean values were D2% = 2.36 Gy (range, 2.13–2.59 Gy), D50% = 2.06 Gy (range, 2.03–2.09 Gy), and D98% = 1.87 Gy (range, 1.83–1.91 Gy).

Across all treatments and fractionation schemes, the mean homogeneity index (HI = D2%/D98%) was 1.24 (range, 1.11–1.41). The average percentage of PTV volume receiving ≥ 110% and ≥ 115% of the prescribed dose was 1.53% (range, 0.03–5.34%) and 0.32% (range, 0.00–3.04%), respectively, across all treatment schedules. Summary of dosimetric parameters are included in Table 3.

**Table 3:** Dosimetric results by fractionation scheme

Dosimetric parameter	12 Gy (6 × 2 Gy)	4 Gy	2 Gy
D2% (Gy)	13.32 (12.90–14.76)	4.23	2.36 (2.13–2.59)
D50% (Gy)	12.55 (11.95–13.84)	4.04	2.06 (2.03–2.09)
D98% (Gy)	10.89 (9.69–12.60)	3.75	1.87 (1.83–1.91)
Homogeneity Index (HI)	Mean 1.24 (range, 1.11–1.41)		
PTV volume ≥ 110% (%)	Mean 1.53 (range, 0.03–5.34)		
PTV volume ≥ 115% (%)	Mean 0.32 (range, 0.00–3.04)		

In patients treated with the 12 Gy regimen (24/27 patients), the mean PTV dose was 12.36 Gy (range, 11.50–13.76 Gy). Mean, maximum, and minimum doses to organs at risk are summarized in Table 4.

**Table 4:** Doses to organs at risk in patients treated with the 12 Gy regimen

Organ	Mean Dose (Gy)	Range (Gy)
Lungs mean	10.12	8.97 – 11.07
Lungs maximum	12.80	11.41– 14.33
Lungs minimum	7.93	5.93 – 9.44
Kidney mean	10.92	9.29 – 13.03
Kidney maximum	12.78	11.29 – 14.44
Kidney minimun	9.38	5.35 – 11.37

### Treatment session

Image guidance was employed in all treatment sessions. A total of 117 sessions were performed using kilovoltage–megavoltage (kV–MV) imaging, and 31 sessions used extended field cone-beam computed tomography (CBCT).

The mean displacements applied were 0.06 cm lateral (range 0.00–2.00 cm), 0.26 cm vertical (0.00–2.00 cm), and 0.03 cm longitudinal (0.00–1.30 cm) in head-first plan. Laterally, 0.26 cm (range: 0.00–2.00 cm) vertically, and 0.03 cm (range: 0.00–1.30 cm) longitudinally in feet first plan.

Fractional treatment delivery time ranged from 60 to 159 min, with a mean of 87 min. The longest component of the session was the image acquisition and registration process. Specifically, IGRT using kV–MV imaging required an average of 14 min (range, 4–41), whereas CBCT-based verification took approximately 10 min per hemibody (range, 6–17).

### Clinical outcomes

All planned treatment sessions were completed without interruptions or unscheduled discontinuities. Of the 27 patients included in the analysis, 26 (96.3%) completed treatment as planned. One patient died during therapy from respiratory sepsis secondary to grade 4 neutropenia resulting from prior chemotherapy.

Two patients (7.4%) developed veno-occlusive disease, and one patient (3.7%) developed interstitial pneumonitis as severe acute toxicities.

At a median follow-up of 8 years, disease relapse was observed in 4 patients (15.4%), whereas 23 patients (85.2%) remained disease-free. Sixteen patients (61.6%) were alive, and 11 patients (40.7%) had died at the time of analysis.

## Discussion

Total body irradiation (TBI) constitutes an essential component of hematopoietic stem cell transplantation. Both the treatment schedule and delivery technique have evolved significantly in recent years. Over the past two decades, clinical practice has shifted from 2 and 3D open-field techniques to volumetric approaches. In earlier methods, patients were simulated in the supine position but treated using direct fields while seated or in the lateral decubitus position. More recently, volumetric techniques, initially helical tomotherapy (HT) and subsequently volumetric modulated arc therapy (VMAT), have redefined treatment planning, delivery, and quality-assurance standards.

The adoption of CT-based planning with the patient in the treatment position marked a pivotal advance toward conformal whole-body irradiation. Helical tomotherapy, introduced in 2005, was the first commercial platform capable of continuous modulation over lengths exceeding 150 cm, and was rapidly endorsed by international guidelines. However, limited access to these specialized systems prompted the development of VMAT-based TBI using conventional LINACs. Early implementations were reported by Springer et al. in 2016 [9], followed by multicenter studies published 2019 and 2025.

This study highlights the clinical utility, feasibility, and dosimetric benefits of VMAT- TBI delivered on a conventional LINAC as an optimized conditioning approach for hematopoietic stem cell transplantation (HSCT). Oven recent years, the incorporation of new technologies has led to a progressive evolution in the TBI technique, culminating in the protocol presented in this work.

Modern TBI aims to achieve a uniform dose distribution across the planning target volume (PTV) while minimizing exposure to organs at risk (OARs). These elements are crucial for reducing treatment-related toxicity and improving patient clinical outcomes.

In our cohort, the mean PTV dose in the 12 Gy fractionation was 12.36 Gy (range: 11.50–13.76 Gy) with a HI of 1.24 (range: 1.11–1.41). This compares favorably to HI 1.08 and 1.07 [30, 31] for HT and is reasonably close to HI 1.16 reported for conventional LINAC TBI [21].

The observed D2% (13.32 Gy, range 12.90–14.76 Gy) and D98% (10.89 Gy, range: 9.69–12.60 Gy) values align with the dosimetric constraints recommended by international guidelines, suggesting adequate target coverage while avoiding significant dose escalation in small subvolumes [2, 32]. Moreover, regions receiving 110% and 115% of the prescribed dose represented only 1.53% (range: 0.03–5.34%) and 0.32% (range: 0.00–3.04%) of the total PTV, respectively. This minimal presence of hot spots supports the robustness of our planning methodology and reinforces the capacity of VMAT-TBI to reduce toxicities associated with dose inhomogeneities [27].

Patient positioning and immobilization play a crucial role in treatment accuracy and reproducibility. Displacements below 5 mm are generally considered clinically acceptable [33, 34], and in our study, the mean displacements (0.06 cm lateral, 0.26 cm vertical, and 0.03 cm longitudinal) demonstrate excellent reproducibility between sessions. The introduction of extended field cone-beam computed tomography (CBCT) in 2023 further enhanced setup accuracy. It also reduced image-guidance time and minimized inter-fraction variability. This represented an important step toward greater workflow efficiency and improved patient comfort.

The mean treatment session duration was 87 min, which compares favorably with times reported in other VMAT and HT series [16, 32, 35]. In the Texas cohort, the mean duration was 72 min (range 33–147) [16]. The integration of extended-field CBCT reduced verification time, streamlining clinical workflow. It should be noted that our reported times were measured from the first image acquisition to the completion of the final field. Therefore, approximately 10 additional minutes per session should be considered for initial patient setup. Longer durations were observed during the first sessions, likely due to patient unfamiliarity with the immobilization system. As sessions progressed, setup became faster and more reproducible.

Our preliminary clinical outcomes indicate that VMAT-TBI was well tolerated. A total of 26 out of 27 patients (96,3%) completed treatment without interruptions. The use of scheduled premedication before each radiotherapy session, accurate and reproducible immobilization, and reduced treatment times likely contributed to this high level of tolerability. In addition, delivering radiotherapy prior to intensification chemotherapy may have further supported treatment completion.

Radiation-induced pulmonary toxicity remains a major concern in TBI, particularly in myeloablative regimens. Previous studies using conventional TBI techniques reported lung dose heterogeneities exceeding 10–20% [2, 7, 14], predisposing patients to pneumonitis and fibrosis. In our study, the mean lung dose was 10.12 Gy (range: 8.97–11.07 Gy), with a maximum of 12.80 Gy (range: 11.41–14.33 Gy), complying with the recommended constraint of < 12 Gy while ensuring adequate target coverage. Only one patient (3.7%) in our cohort developed interstitial pneumonitis, aligning with reported VMAT-TBI incidences of 0–9% [36]. This reduction in pulmonary dose explains the decrease in pneumonitis rates from 12% with 2D TBI to 2% with VMAT reported in a recent series [36].

The relapse rate in our study was 14.81%, notably lower than the 28–33% reported for conventional TBI in HSCT [37, 38]. Zhang-Velten et al. documented 1- and 2-year overall survival rates of 90% and 79%, respectively, which are consistent with our preliminary findings [16]. However, long-term follow-up is needed to assess whether these dosimetric and technical improvements translate into superior overall and disease-free survival compared to historical cohorts.

A major challenge in implementing TBI is the extended occupancy of the LINAC, which adds logistical complexity. As a result, these treatments require meticulous coordination to ensure feasibility within standard clinical workflows. Initially, the lack of appropriate software to fuse the upper and lower CT datasets necessitated collaboration with the Nuclear Medicine Department. At that time, the PET/CT scanner was the only system capable of acquiring whole-body images in a single acquisition. The subsequent implementation of CTs concatenation tools resolved this limitation, enabling fully independent treatment planning within the Radiation Oncology Department. This improvement significantly enhanced workflow efficiency and autonomy.

The integration of advanced tools, such as extended-field CBCT and CT concatenation, has enabled the optimization of complex TBI procedures, reducing treatment times, improving patient compliance, and enhancing the utilization efficiency of conventional linear accelerators. The ability to achieve highly conformal dose distributions while minimizing irradiation of OAR is expected to improve the therapeutic ratio.

Large randomized multicenter trials comparing VMAT-TBI with historical 2D/3D approaches are unlikely to be feasible. Centers with access to conformal techniques will progressively phase out older methods. Therefore, it is essential for institutions performing VMAT-TBI to report their clinical experience. This will help refine workflows, harmonize treatment parameters, and expand access to conformal TBI in regions without helical tomotherapy. Observational studies and collaborative registries may help to evaluate potential differences in graft-versus-host disease incidence and relapse-free survival, while acknowledging that TBI is only one of several factors influencing GvHD. Delivering total body irradiation using conventional linear accelerators may broaden access to this treatment modality and promote collaborative research on dose regimens and treatment optimization at both national and international levels. Nevertheless, TBI should continue to be performed in reference centers with adequate expertise and experience to ensure safe and effective treatment delivery.

This study has several limitations.

First, its retrospective nature limits the ability to draw causal conclusions.

Second, the cohort size is relatively small, reflecting the limited number of TBI candidates in our region.

Third, a relevant weakness of the workflow was the initial lack of a dedicated and standardized solution for whole-body CT concatenation once standalone CT acquisitions were introduced. Although full-body imaging was initially available through PET-CT scans, the subsequent use of CT images from our department required the development and validation of a concatenation strategy. This resulted in a labor-intensive and iterative process in which several methods were explored, including open-source software and commercial platforms not specifically designed for automated whole-body CT stitching, such as Velocity™. Consequently, early implementations required extensive and time-consuming quality control procedures to verify anatomical continuity, registration accuracy, and consistency of Hounsfield Unit values, increasing workload and hindering the establishment of a fully standardized workflow.

This limitation was subsequently mitigated through the implementation of a dedicated CT concatenation script (TXIhelper)^(25)^ provided and recommended by Varian. The tool, developed using the Eclipse Scripting Application Programming Interface (ESAPI) and integrated within the Eclipse External Beam Planning environment, enables automated concatenation of a head-first supine superior CT series with a feet-first supine inferior CT series following rigid registration. The resulting output is a single, fully merged CT dataset that preserves HU integrity and anatomical continuity.

## Conclusion

Our study demonstrates that VMAT-based TBI is a viable and advantageous alternative to conventional 2D-TBI, providing improved dose homogeneity, enhanced organ sparing and high reproducibility, comparable to that achieved with HT systems.

These results are promising and support the use of VMAT techniques on conventional LINACs for TBI treatments. Further prospective studies with larger cohorts and long-term follow-up are warranted to confirm the clinical advantages and long-term outcomes associated with this approach.

In addition, this protocol demonstrates that VMAT-TBI can be safely implemented in centers lacking helical tomotherapy, providing broader access to conformal TBI in resource-limited settings.

## Declarations

### Data availability

The data supporting the findings of this study are not publicly available due to confidentiality and data protection considerations.
